# Using 3D Convolutional Neural Networks for Tactile Object Recognition with Robotic Palpation

**DOI:** 10.3390/s19245356

**Published:** 2019-12-05

**Authors:** Francisco Pastor, Juan M. Gandarias, Alfonso J. García-Cerezo, Jesús M. Gómez-de-Gabriel

**Affiliations:** Robotics and Mechatronics Group, University of Málaga, 29071 Málaga, Spain; jmgandarias@uma.es (J.M.G.); ajgarcia@uma.es (A.J.G.-C.); jesus.gomez@uma.es (J.M.G.-d.-G.)

**Keywords:** tactile perception, robotic palpation, underactuated grippers, deep learning

## Abstract

In this paper, a novel method of active tactile perception based on 3D neural networks and a high-resolution tactile sensor installed on a robot gripper is presented. A haptic exploratory procedure based on robotic palpation is performed to get pressure images at different grasping forces that provide information not only about the external shape of the object, but also about its internal features. The gripper consists of two underactuated fingers with a tactile sensor array in the thumb. A new representation of tactile information as 3D tactile tensors is described. During a squeeze-and-release process, the pressure images read from the tactile sensor are concatenated forming a tensor that contains information about the variation of pressure matrices along with the grasping forces. These tensors are used to feed a 3D Convolutional Neural Network (3D CNN) called 3D TactNet, which is able to classify the grasped object through active interaction. Results show that 3D CNN performs better, and provide better recognition rates with a lower number of training data.

## 1. Introduction

Recent advances in Artificial Intelligence (AI) have brought the possibility of improving robotic perception capabilities. Although most of them are focused on visual perception [1], existing solutions can also be applied to tactile data [2,3,4]. Tactile sensors measure contact pressure from other physical magnitudes, depending on the nature of the transducer. Different types of tactile sensors [5,6,7,8,9] have been used in robotic manipulation [10,11] for multiple applications such as slippage detection [12,13], tactile object recognition [14,15], or surface classification [16,17], among others.

Robotic tactile perception consists of the integration of mechanisms that allow a robot to sense tactile properties from physical contact with the environment along with intelligent capacities to extract high-level information from the contact. The sense of touch is essential for robots the same way as for human beings for performing both simple and complex tasks such as object recognition or dexterous manipulation [18,19,20]. Recent studies focused on the development of robotic systems that behaves similar to humans, including the implementation of tactile perception capabilities [21,22]. However, tactile perception is still a fundamental problem in robotics that has not been solved so far [23]. In addition, there are multiple applications, not limited to classic robotic manipulation problems that can benefit from tactile perception such as medicine [24], food industry [3], or search-and-rescue [4], among others.

Many works related to tactile perception use pressure images after the interaction [25], which means that the interaction is considered static or passive. However, tactile perception in the real world is intrinsically active [26]. A natural or bio-inspired haptic Exploratory Procedure (EP) for perceiving pressure or stiffness of an object must consider dynamic information [27]. According to [28], the haptic attributes that can be perceived depends on the EP.

A survey on the concept of active tactile perception considering biological and psychological terms is presented in [29]. In this chapter, and according to [30], two approaches for tactile perception in robots are possible: perception for action, which means that the perceived information is used to guide the robot (i.e., dexterous manipulation and grasp control), and action for perception, which means that the robot explores the environment to collect data (i.e., active perception and haptic exploration). Hence, an active tactile perception approach can be defined as one in which the data are collected during an active EP using an active sensing approach (e.g., tactile sensing). This means that action and perception are not separated, and the robot collects dynamic data depending on the action, while this action is occurring. Therefore, although both static and dynamic tactile data are useful for many robotic applications, it can be considered that active perception is more faithful to the real sense of touch, and the information acquired using active tactile sensing reflects the attributes of the grasped objects better. Static pressure images only contain information about stiffness and shape of the object when a certain force is applied [14], while the changes of the pressure distribution over force contain information about the variation of shape and stiffness during the whole EP [31]. This dynamic information allows us to distinguish both rigid and deformable objects [32].

**Figure 1:**
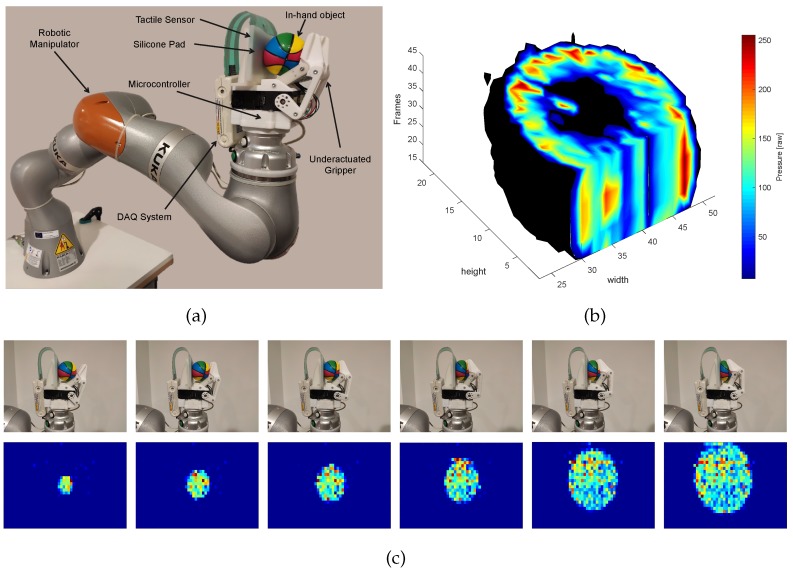
The full experimental system formed by a robotic manipulator, an underactuated gripper with a tactile sensor, and the control electronics (**a**), a 3D tensor representation of active tactile information when the gripper is grasping a squeezable ball (**b**), and a subset of pictures and their respective tactile images of a grasping sequence of another squeezable ball (**c**). In (**b**), the tensor is sectioned to show the intrinsic attributes and pressure variations of the grasped object.

This paper addresses the shortcomings mentioned above and is focused on the active tactile perception problem in robotics. A robotic palpation process with active tactile sensing, based on a squeeze-and-release motion for distinguishing grasped objects, both rigid and deformable, is presented (see Figure 1). The robotic EP conceives a novel representation of dynamic tactile information based on sequences of pressure images and an AI method based on 3D Convolutional Neural Networks (3D CNNs) for active tactile perception. A tactile sensor array is integrated into the thumb of a gripper with two underactuated fingers to get sequences of tactile images. These sequences are represented as 3D tensors similar to Magnetic Resonance Imaging (MRI). However, in this case, 3D tactile tensors represent the variation of pressure distribution over applied force, whereas MRI contains information about cross-sectional images of internal structures and organs over distance. Although the type of information contained in MRIs and 3D tactile tensors is different, methods such as 3D CNNs used to process MRI information [33,34] might be used for tactile data with good results in this application as we explored in our previous work [35]. In this work, our preliminary study is expanded: a high-resolution tactile sensor has been integrated into a new gripper where the palpation process (e.g., the EP) is fully autonomous, so the robot controls the grasping force. As a result, not only objects with different elasticity are compared and classified, but also objects that contain internal inclusions and bags of objects which provide different pressure images each time, have been tested. In particular, 24 objects have been used: rigid, deformable, and in-bag; and the results are compared against 2D CNN-based methods. Altogether, the main contribution of this paper relates to the entire process of active tactile perception, considering the use of an underactuated, sensorized gripper to carry out the EP, and a 3D CNN for tactile perception.

The relevance of this contribution relies on different factors. First, the presented method achieves better performance in the discrimination problem for all kinds of objects, and, in case the number of classes increases, a lower number of training data are needed to obtain higher accuracy rates than classic 2D networks. Second, it is also shown that, in case of misclassification, the resulting object class has almost indistinguishable physical features (e.g., soda cans of different capacities), where 2D CNNs, in the event of failure, give disparate output classes unrelated to the class of the grasped object.

This paper is structured as follows: In Section 2, the current state-of-the-art related to this topic is introduced. In Section 3, the underactuated gripper and the 3D CNN-based method used for tactile perception are described. The experimental protocol and results are explained in Section 4, and a thorough and detailed discussion of our results in comparison with related works is presented in Section 5. Finally, the conclusion and future research lines are exposed in Section 6.

## 2. Related Work

Related works within the scope of tactile perception in robotics focus on tactile object-recognition from pressure-images, deep-learning methods based on CNNs, and active tactile perception.

### 2.1. Tactile Object Recognition

Two main approaches for tactile object recognition may be considered depending on the nature of the EP: On one hand, perceiving attributes from the material composition, which are typically related to superficial properties like roughness, texture, or thermal conductivity [36,37,38]. On the other hand, other properties related to stiffness and shape may also be considered for object discrimination [39,40,41]. Most of these works are based on the use of novel machine learning-based techniques. That way, different approaches can be followed, such as Gaussian Processes [42], k-Nearest Neighbour (kNN) [25], Bayesian approaches [43], k-mean and Support Vector Machines (SVM) [44], or Convolutional Neural Networks (CNNs) [45], among others. Multi-modal techniques have also been considered in [46], where they demonstrated that considering both haptic and visual information generally gives better results.

### 2.2. Tactile Perception Based on Pressure Images

Concerning the latter approach, most of the existing solutions in literature acquire data from tactile sensors, in the form of matrices of pressure values, analog to common video images [47]. In this respect, multiple strategies and methodologies can be followed. In [25], a method, based on *Scale Invariant Feature Transform* (SIFT) descriptors, is used as a feature extractor, and the kNN algorithm is used to classify objects by their shape. In [15], Luo et al. proposed a novel multi-modal algorithm that mixes kinesthetic and tactile data to classify objects from a 4D point cloud where each point is represented by the 3D position of the point and the pressure acquired by a tactile sensor.

### 2.3. CNNs-Based Tactile Perception

One recent approach for tactile object discrimination consists of the incorporation of modern deep learning-based techniques [48,49]. In this respect, the advantages of Convolutional Neural Networks (CNNs) such as translational and rotational invariant property enable the recognition in any pose [50]. A CNN-based method to recognize human hands in contact with an artificial skin has been presented in [44]. The proposed method benefits from the CNN’s translation-invariant properties and is able to identify whether the contact is made with the right or the left hand. Apart from that, the integration of the dropout technique in deep learning-based tactile perception has been considered in [49], where the benefits of fusing kinesthetic and tactile information for object classification are also described, as well as the differences of using planar and curved tactile sensors.

### 2.4. Active Tactile Perception

In spite of the good results obtained by existing solutions in tactile object recognition, one of the main weaknesses is that most of these solutions only consider static or passive tactile data [25]. As explained, static tactile perception is not a natural EP to perceive attributes like pressure or stiffness [27]. Pressure images only have information about the shape and pressure distribution when a certain force is applied [14]. On the other hand, sequences of tactile images also contain information about the variation of shape (in the case of deformable objects [32]), stiffness, and pressure distribution over time [31].

Time-series or sequential data are important to identify some properties. This approach has been followed in some works for material discrimination [51,52]. In [53], an EP is carried out by a robotic manipulator to get dynamic data using a 2D force sensor. The control strategy of the actuator is critical to apply a constant pressure level and perceive trustworthy data. For this purpose, a multi-channel neural network was used achieving high accuracy levels.

Pressure images obtained from tactile sensors have also been used to form sequences of images. In [3], a flexible sensor was used to classify food textures. A CNN was trained with sequences of tactile images obtained during a food-biting experiment in which a sensorized press is used to crush food, simulating the behavior of a mouth biting. The authors found that the results when using the whole biting sequence or only the first and last tactile images were very similar because the food was crushed when a certain level of pressure was applied. Therefore, the images before and after the break point were significantly different. For other applications, as it was demonstrated in [54], Three-Dimensional Convolutional Neural Networks (3D CNNs) present better performance when dealing with sequences of images than common 2D CNNs.

## 3. Materials and Methods

The experimental setup is composed of a gripper with a tactile sensor. The gripper, the representation of 3D tactile information, and the 3D CNN are described next.

### 3.1. Underactuated Gripper

**Figure 2:**
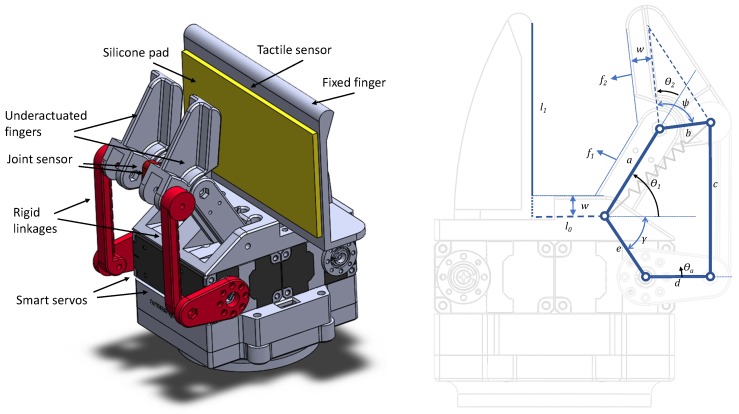
Gripper design (**left**) with two independent underactuated fingers and one fixed thumb with a tactile sensor covered with a silicone pad. The kinematic structure of the underactuated fingers (**right**) shows the five-bar structure with associated parameters and degrees of freedom (DOFs).

The active perception method has been implemented using a gripper with two parallel underactuated fingers and a fixed tactile-sensing surface (see Figure 2). The reason for using an underactuated gripper is that this kind of gripper allows us to apply evenly spread pressure to the grasped objects, and the fingers could adapt to their shape, which is especially useful when grasping deformable or in-bag objects. In our gripper, each underactuated finger has two phalanxes with two (DOFs) $\theta_{1}$ and $\theta_{2}$, and a single actuator $\theta_{a}$ capable of providing different torque values $\tau_{a}$. The values of the parameters of the kinematics are included in Table 1. A spring provides stiffness to the finger to recover the initial position when no contact is detected.

Two smart servos (Dynamixel XM-430 from ROBOTIS (Seoul, Korea)) have been used to provide different torques trough rigid-links, using a five-bar mechanical structure to place the servos away from the first joint. Thus, the relationship between $\tau_{a}$ and the joint torques ($\tau_{1}$, $\tau_{2}$) can be expressed as a transfer matrix T, and the computation of the Cartesian grasping forces ($f_{1}$, $f_{2}$) from the joint torques is defined by the Jacobian matrix F=J(θ)τ.

The computation of those matrices requires knowledge of the actual values of the underactuated joints. For this reason, a joint sensor has been added to the second joint of each finger. The remaining joint can be computed as the actual value of the servo joint, which is obtained from the smart servos. Two miniature potentiometers (*muRata* SV01) have been used to create a special gripper with both passive adaptation and proprioceptive feedback.

The dynamic effects can be neglected when considering slow motions and lightweight fingers. This way, a kinetostatic model of the forces can be derived in Equation (1) as described in [55]:

(1) $$F=J{\left(\theta \right)}^{-T}\;T{\left(\theta \right)}^{-T}\;\tau .$$

Although the actual Cartesian forces could be computed, each object with a different shape should require feedback control to apply the desired grasping forces. In order to simplify the experimental setup, an open-loop force control has been used for the grasping operations, where the actuation (pulse-width modulation - PWM) of the direct current (DC) motors of the smart servos follows a slow triangular trajectory from a minimum value (5%) to a maximum (90%) of the maximum torque of 1.4 N.m of each actuator. The resulting position of each finger depends on the actual PWM and the shape and impedance of each contact area.

Finally, a microcontroller (Arduino Mega2560) has been used to acquire angles form the analog potentiometers and communicating with the smart servos in real time, with a 50 ms period.

### 3.2. Tactile Sensor

A Teskcan (South Boston, MA, USA) sensor model 6077 has been used. This high-resolution tactile-array has 1400 tactels (also called taxels o sensels), with 1.3×1.3 mm size each. The sensor presents a density of $27.6\;\mathrm{tactels}/{\mathrm{cm}}^{2}$ distributed in a 28×50 matrix. The main features of the sensor are presented in Table 2. The setup includes the data acquisition system (DAQ) (see Figure 1a), and the Tekscan real-time software development kit (SDK) (South Boston, MA, USA).

A silicone pad of 3 mm has been added to the tactile sensor to enhance the grip and the image quality, especially when grabbing rigid objects. In particular, the ${\mathrm{Ecoflex}}^{TM}00-30$ rubber silicone has been chosen due to its mechanical properties.

### 3.3. Representation of Active Tactile Information

As introduced in Section 1, a natural palpation EP to get information about the stiffness of an in-hand object is dynamic. In this respect, it seems evident that a robotic EP should also be dynamic so that the information acquired during the whole squeeze-and-release process describes the external and internal tactile attributes of an object.

The pressure information can be represented in multiple ways, commonly as sequences of tactile images. However, in this case, a more appropriate structure is in the form of 3D tactile tensors. An example of this type of representation is presented in Figure 1b, which is similar to MRI, except that, in this case, the cross-sectional images contain information about the pressure distribution at the contact surface for different grasping forces.

**Figure 3:**
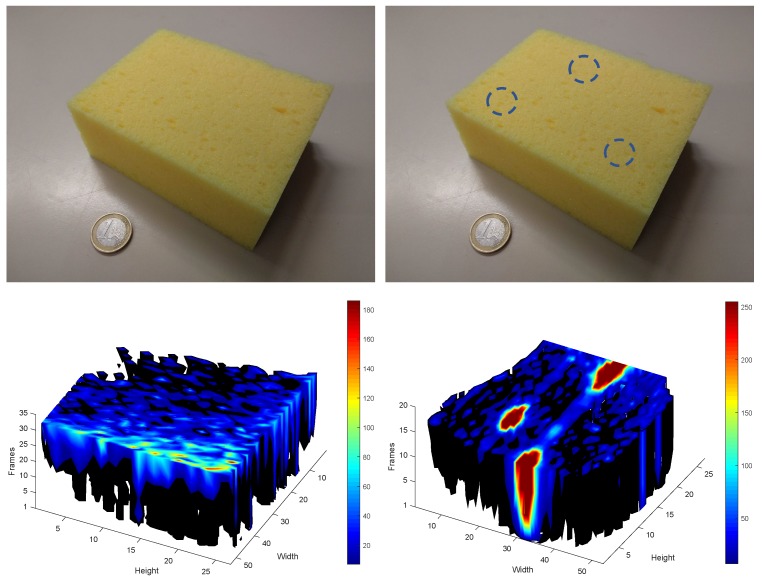
3D tactile tensors (**bottom**) of the same sponge with and without hard inclusions (**top**). The inclusions become visible as grasping force increases but cannot be seen in the picture of the sponge.

To show the advantages of 3D tactile tensors, sectioned tensors of the same sponge, with and without hard inclusions, are shown in Figure 3. The inclusions become perfectly visible as the grasping force increases.

### 3.4. 3D TactNet

When using 3D tactile information, it is necessary to control the applied forces to obtain a representative pressure-images from a certain object. For 3D CNNs, each tensor has information about the whole palpation process. On the other hand, when dealing with soft or shape-changing objects, this operation is more challenging using 2D CNNs, as a high amount of training data would be necessary, or selected data captured at optimal pressure levels, which also depends on the stiffness of each object.

In previous works, we trained and validated multiple 3D CNNs with different structures and hyperparameters to discriminate deformable objects in a fully-supervised collection and classification process [35]. Here, although the classification is still supervised, the grasping and data collection processes have been carried out autonomously by the robotic manipulator. According to the results of our previous work, the 3D CNN with the highest recognition rate, and compatible with the size of the 3D tensors read from our tactile sensor, was a neural network with four layers, where the first two were 3D convolutional, and the last two were fully connected layers. The network’s parameters have been slightly modified to fit a higher number of classes and to adjust the new 3D tensor, which has a dimension of $\left[28\times 50\times 51\right]$.

**Figure 4:**
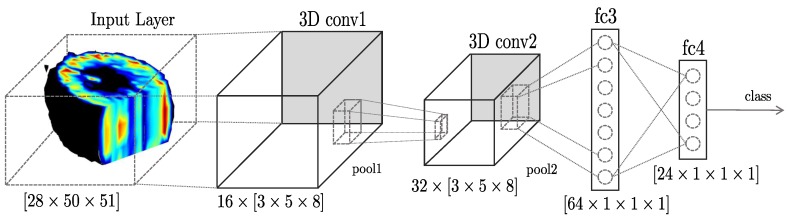
Architecture of TactNet3D, which is formed by four layers, the first two are 3D convolutional layers with kernel sizes $16\times \left[5\times 3\times 8\right]$ and $32\times \left[5\times 3\times 8\right]$, respectively, and two fully connected layers with 64 and 24 neurons, respectively.

The architecture of this network, called TactNet3D, is presented in Figure 4. This network has two 3D convolutional layers $\left(C=\left[{3D\mathrm{conv}}_{1}\;,{3D\mathrm{conv}}_{2}\right]\right)$ with kernels $16\times \left[3\times 5\times 8\right]$ and $32\times \left[3\times 5\times 8\right]$, respectively, and two fully connected layers $\left(F=\left[{\mathrm{fc}}_{3},{\mathrm{fc}}_{4}\right]\right)$ with 64 and 24 neurons, respectively. Each convolutional layer also includes a Rectified Linear Unit (ReLU), batch normalization with $\epsilon =10^{-5}$, and max-pooling with filters and stride equal to 1. In addition, ${\mathrm{fc}}_{3}$ incorporated a dropout factor of 0.7 to prevent overfitting. Finally, a softmax layer is used to extract the probability distribution of belonging to each class. The implementation, training, and testing of this network has been done using the Deep Learning Toolbox in Matlab (R2019b, MathWorks,Natick, MA, USA).

## 4. Experimental Protocol and Results

This section presents the procedure for the dataset collection and the experiments. The dataset is conformed by three subsets of data: Rigid, deformable, and in-bag objects, which are described in more detailed below. Similarly, four experiments have been carried out to show the performance of the method and compare the results of dynamic and static methods: Experiment 1 for rigid objects, experiment 2 for deformable objects, experiment 3 for in-bag objects, and experiment 4 for the whole dataset.

### 4.1. Dataset

#### 4.1.1. Collection Process

The dataset collection process consists of capturing sequences of tactile images and creating a 3D tactile tensor. For this purpose, the underactuated gripper holds an object and applies incremental forces while recording images over the whole palpation process. Each object, depending on its internal physical attributes, has a unique tactile frame for each amount of applied force. The dataset collection has been carried out by the gripper, recording 51 tactile frames per squeeze. This process is made by the two active fingers of the gripper, which are moved by the two smart servos in torque control mode with incremental torque references. Finally, 1440 3D tactile tensors have been obtained, for a total of 24 objects with 60 tactile tensors each. In Figure 1c, a grasping sequence is shown. The sequence at the top, from the left to the right, shows the grasping sequence due to the progressive forces applied by the underactuated gripper to the ball 2, and the sequence at the bottom, from the left to the right, shows the tactile images captured by the pressure sensor.

For machine learning methods, it is important to have the greatest possible variety in the dataset. In order to achieve this goal, the incremental torque is increased in random steps, so that the applied forces between two consecutive frames are different in each case. This randomness is also applied due to the intention to take a dataset that imitates the palpation procedure that could be carried out by a human, in which the exact forces are not known. Another fact that has been considered for the dataset collection process is that the force is applied to the object through the fingers of the gripper; therefore, non-homogeneous pressure is exerted on the whole surface of the object. Therefore, in order to obtain all of the internal features of the objects, multiple grasps with random positions and orientations of the objects have been obtained.

#### 4.1.2. Rigid Objects

**Figure 5:**
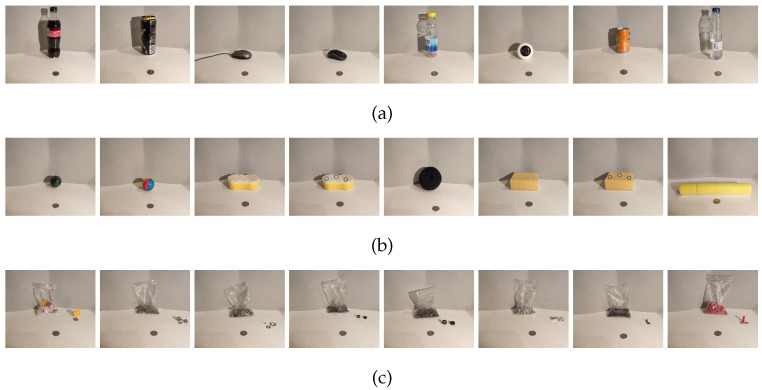
Pictures of the 24 objects used in experiments. Rigid objects (**a**), from left to right: bottle of coke, energy drink can, mouse 1, mouse 2, bottle of ice tea, skate wheel, soda can, and bottle of water. Deformable objects (**b**), from left to right: ball 1, ball 2, sponge rough, sponge rough with inclusions, sponge scrunchy, sponge soft, sponge soft with inclusions, and sponge pipe. In-bag objects (**c**), from left to right: gears, mixed nuts, mixed washer, M6 nuts, M8 nuts, M10 nuts, rivets, and rubber pipes.

Eight objects of the dataset are considered as rigid because they barely change their shape when the gripper tightens them. The rigid dataset is composed of subsets of objects with similar features (e.g., the subset of bottles and the subset of cans) which are very different from each other. The subset of rigid objects is shown in Figure 5a.

#### 4.1.3. Deformable Objects

Another subset of the dataset is the deformable objects. This subset consists of eight objects that change his initial shape substantially when a pressure is applied over it but recover its initial shape when the pressure ends. This subgroup also has objects with similar elasticity (e.g., balls and sponges). The set of deformable objects is shown in Figure 5b.

#### 4.1.4. In-Bag Objects

The last subset of objects included in the dataset is composed of plastic bags with a number of small objects. Bags are shuffled before every grasp, so that the objects in the bag are placed in different positions and orientations. Hence, the tactile images are different depending on the position of the objects. Another characteristic of this group is that in-bag objects may change their position randomly during the grasping process. As in the other subgroups, bags with similar objects have been chosen (e.g., M6, M8, or M10 nuts). In-bag objects are shown in Figure 5c.

### 4.2. Experiments and Results

According to [45], three approaches can be followed to classify tactile data with 2D CNNs: training the network from scratch (method 1), using a pre-trained network with standard images and re-training the last classification layers (method 2), or changing the last layers by another estimator (method 3). The best results for each approach were obtained by TactNet6, ResNet50_NN, and VGG16_SVM, respectively. In this work, four experiments have been carried out to validate and compare the performance of TactNet3D against these 2D CNN structures considering only the subset of rigid objects, the subset of deformable objects, the subset of in-bag objects, and the whole dataset. The training, validation, and test sets to train the 2D CNN-based methods are formed using the individual images extracted from the 3D tactile tensors.

**Figure 6:**
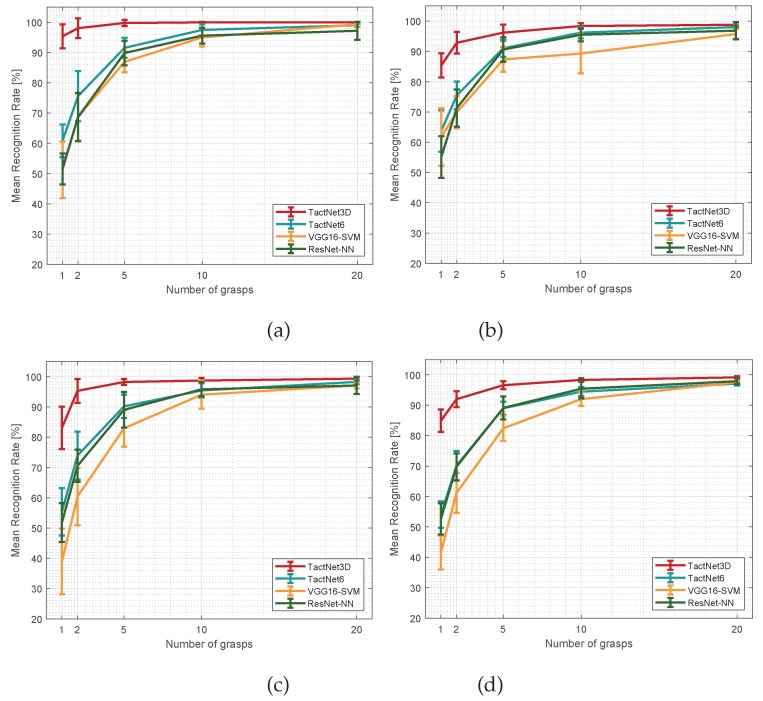
Experimental results of the experiment with rigid objects (**a**), deformable objects (**b**), in-bag objects (**c**), and all objects (**d**). Error bars represent the standard deviation σ of each recognition rate distribution over a 20-sample testing process.

The performance of each method has been measured in terms of recognition accuracy. Each network has been trained 20 times with each subset, and the mean recognition rate and standard deviation for each set of 20 samples have been compared in Figure 6, where, for each experiment, the results of each method have been obtained using data from 1, 2, 5, 10, and 20 grasps of each object.

**Figure 7:**
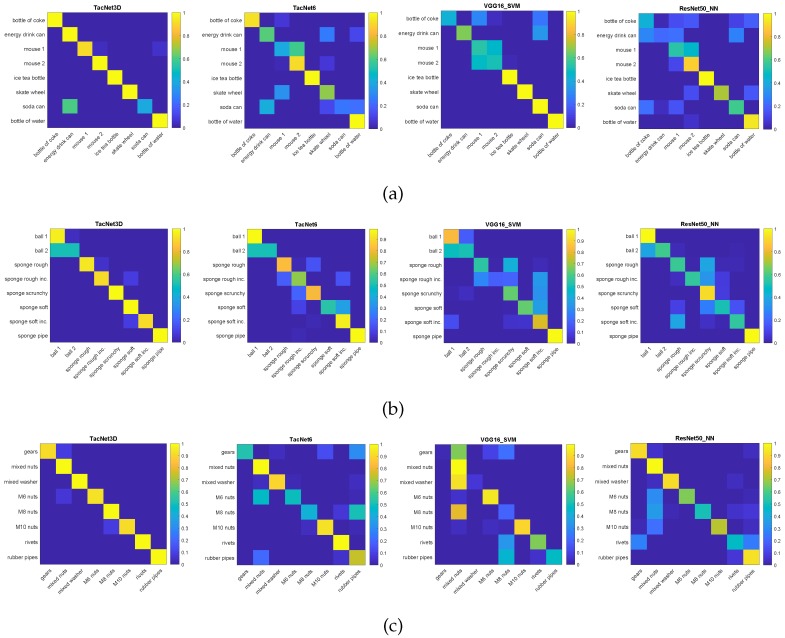
Confusion matrices of the methods, from left to right, TactNet3D, TactNet6, VGG16_SVM and ResNet50_NN, in experiments with rigid objects (**a**), deformable objects (**b**), and in-bag objects (**c**). All of the methods are trained using data from two grasps.

**Figure 8:**
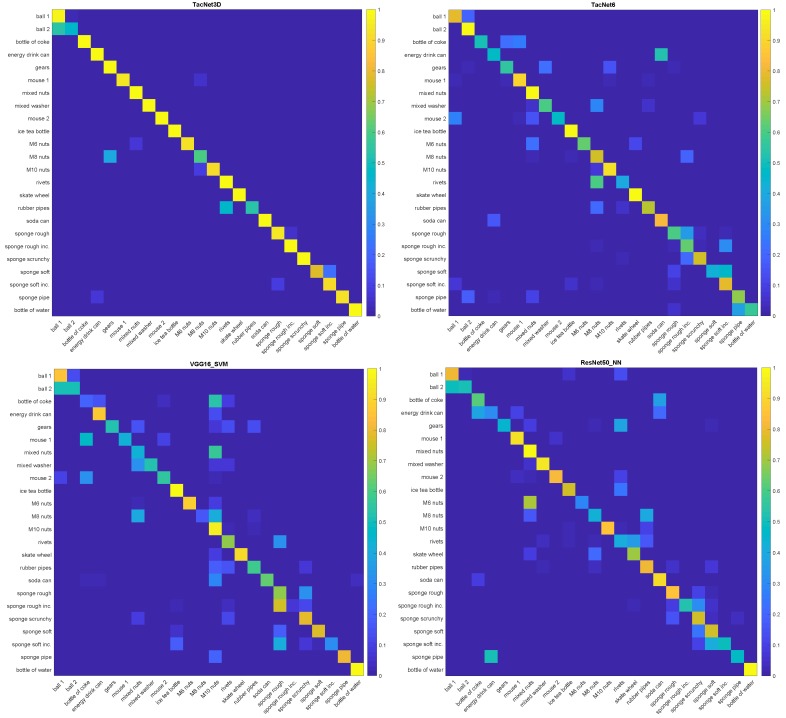
Confusion matrices of the methods, from left to right, TactNet3D, TactNet6, VGG16_SVM and ResNet50_NN, in the experiments with the whole dataset. All the methods are trained using data from two grasps.

Moreover, representative confusion matrices for each method trained in subsets of rigid, deformable, and in-bag objects are presented in Figure 7. In contrast, the confusion matrices related to the whole dataset are presented in Figure 8. These confusion matrices have been obtained for the case in which each method is trained using data from two grasps to show the differences in classification performance.

## 5. Discussion

Regarding the performance of TactNet3D in comparison with 2D CNN-based methods, the results shown in Figure 6 prove that the recognition rate of the first one is better than the latter in all the studied cases. For all kinds of objects, rigid, deformable, or in-bag, and all the amount of grasps used as training data, TactNet3D outperforms 2D CNNs.

In addition, the differences in classification accuracy are higher when the number of training data are lower, getting better results when training TactNet3D with one or two grasps than 2D CNNs with five or ten grips in some cases. Therefore, it is not only shown that the performance is better, but also the adaptability of TactNet3D as the amount of data needed to train the network is lower, which is especially interesting for online-learning.

In addition, in the misclassification cases, the resulting object class given by TactNet3D has almost indistinguishable physical features to those of the grasped object, unlike 2D CNNs, which may provide disparate results, as can be seen in the confusion matrices presented in Figure 7 and Figure 8. Looking at some object subsets with similar physical features such as the sponges, the different bag of nuts or the cans, it can be observed that the output given by TactNet3D corresponds to objects form the same subset, whereas 2D CNN output classes of objects with different features in some cases (e.g., bottle of coke and M10 nuts in Figure 8 bottom left). This phenomenon is interesting from the neurological point of view of an artificial touch sense as 3DTactNet behaves more similarly to human beings’ sense of touch. However, a broad study of this aspect is out of the scope of this paper and will be considered in future works.

## 6. Conclusions

A novel method for the active tactile perception based on 3D CNN has been presented and used for an object recognition problem in a new robot gripper design. This gripper includes two underactuated fingers that accommodate to the shape of different objects, and have additional proprioceptive sensors to get its actual position. A tactile sensor has been integrated into the gripper, and a novel representation of sequences of tactile images as 3D tactile tensors has been described.

A new 3D CNN has been designed and tested with a set of 24 objects classified in three main categories that include rigid, deformable, and in-bag objects. There are very similar objects in the set, and objects that have changing and complex shapes such as sponges or bags of nuts, in order to assess the recognition capabilities. 3D CNN and classical CNN with 2D tensors have been tested for comparison. Both perform well with high recognition rates when the amount of training data are high. Nevertheless, 3D CNN gets higher performance even with a lower number of training samples, and misclassification is obtained just in very similar classes.

As future works, we propose the use of additional proprioceptive information to train multi-channel neural networks using the kinesthetic information about the shape of the grasped object, along with the tactile images for multi-modal tactile perception. In addition, the use of other dynamic approaches, such as temporal methods (e.g., LSTMs), for both tactile-based and multi-modal-based perception strategies, need to be addressed in more detail. Moreover, a comparison of new active tactile perception methods will be studied in depth.

## Figures and Tables

**Table 1:** Parameter values for the kinematic model of the gripper with underactuated fingers.

Parameter	Value	Parameter	Value
*a*	40 mm	e	27.8 mm
*b*	20 mm	ψ	$90^{\circ }$
*c*	60 mm	γ	$56^{\circ }$
*d*	25 mm	*w*	10 mm
$l_{0}$	25–45 mm	$l_{1}$	70 mm

**Table 2:** Main features of the *Tekscan* 6077 tactile sensor.

Parameter	Value
Max. pressure	34 KPa
Number of tactels	1700
Tactels density	27.6 tactels/cm${}^{2}$
Temperature range	$-40{\;}^{\circ }$C to $+60{\;}^{\circ }$C
Matrix height	53.3 mm
Matrix width	95.3 mm
Thickness	0.102 mm
